# Gender-differences in predictors for time to metabolic syndrome resolution: A secondary analysis of a randomized controlled trial study

**DOI:** 10.1371/journal.pone.0234035

**Published:** 2020-06-25

**Authors:** Seung-Ah Choe, Nan-He Yoon, Seunghyun Yoo, Hyekyeong Kim

**Affiliations:** 1 Department of Obstetrics and Gynecology, School of Medicine, CHA University, Gyunggi-do, Republic of Korea; 2 Department of Health Administration, Hanyang Cyber University, Seoul, Republic of Korea; 3 Department of Public Health Science, Graduate School of Public Health, Seoul National University, Seoul, Republic of Korea; 4 Institute of Health and Environment, Seoul National University, Seoul, Republic of Korea; 5 Department of Health Convergence, Ewha Womans University, Seoul, Republic of Korea; Universidad Miguel Hernandez de Elche, SPAIN

## Abstract

Understanding gender differences in health-related behaviors and their impacts is a crucial aspect of effective primary care. We studied gender-based differences in predictors of metabolic syndrome (MetS) resolution among newly diagnosed MetS patients. This study was a secondary analysis of a prospective clinical trial study comprising of 637 middle-aged and older adults (226 men and 411 women) who underwent a regular health checkup and were newly diagnosed with MetS at 16 different health clinics of 14 metropolitan cities and provinces. We conducted Cox proportional hazard analysis to estimate cumulative probability of MetS resolution within a 12‐month observation period. Among the 637 patients, 47.6% of participants achieved MetS resolution. The resolution rate was similar among men and women (44.7% and 49.1%, respectively, P = 0.320). Low household income (Hazard ratio = 2.62, 95% confidence interval: 1.13–6.08) and current employment (2.29, 1.26–4.13) were associated with a higher cumulative probability of MetS resolution in men than in women. For women, however, longer sleeping hours (1.18, 1.04–1.34) and living with a partner (1.58, 1.06–2.35) were positive predictors of MetS resolution. Being overweight (0.63, 0.44–0.89) was associated with lower cumulative probability of MetS resolution in women than in men. The factors associated with cumulative probability of MetS resolution within the 12-month follow-up were different between men and women. These findings facilitate further exploration on gender-based differences in risk factors for less optimal improvements in MetS.

## Introduction

Metabolic syndrome (MetS) is a clinical condition characterized by a cluster of abnormalities including: hyperglycemia, hypertension, hypertriglyceridemia, reduced high-density lipoprotein cholesterol, and central adiposity [1]. The presence of at least three of the five diagnostic criteria is defined as MetS [2].Metabolic syndrome becomes a global epidemic with the rise in obesity [3].

In several industrialized societies, MetS occur more frequently in women than in men. In United States, the prevalence of MetS is 36.6% in women and 32.8% in men [4]. The prevalence of obesity is greater in African American and Hispanic women than in non-Hispanic whites, suggesting that biological gender and ethnicity play a role in the development of metabolic diseases [5–7]. According to recent reports, 19.2% of men and 27.0% of women in China [8], 32.9% of women and 29.0% of men in Korea [9], 13.4% of women and 10.5% of men in Philippines [10], and 31.5% of women and 25.5% of men in Taiwan [11] are diagnosed with MetS, revealing generally a higher prevalence of MetS in women [12]. The gender difference is generally observed in midlife [11]. Furthermore, the severity of MetS progresses rapidly in middle-aged women due to the menopausal transition [13]. Considering the increasing risk of cardiovascular disease in postmenopausal women, improving the metabolic profile in women with MetS is crucial for reducing mortality and long-term morbidities.

Lifestyle modification or therapeutic lifestyle changes based on behavior therapies are an important and effective strategy for managing newly diagnosed MetS patients [1]. This involves changes in diet, physical activity, and self-monitoring [14]. In the presence of severe obesity with or without uncontrolled hyperglycemia, some researchers suggested metabolic surgery can be effective for a persistent MetS resolution [15, 16]. Evidence exists to support the notion that diet, exercise, and pharmacologic interventions may inhibit the progression of MetS to more severe chronic conditions, such as diabetes mellitus. Although there have been advances of preventive lifestyle interventions, a number of clinical and public health strategies have failed to achieve significant and sustainable changes in affected populations [17, 18]. Considering that perceived support from family members has positive effect on self-efficacy of adults [19], the role of family for lifestyle modifications is crucial as well.

Middle-aged and older women can be more vulnerable to lifestyle-related disorders, due to limited time and economic resources, compared to men in the same age groups [20]. However, men have a higher prevalence of individual MetS components [21]. Therefore, developing effective risk-reducing strategies for women with MetS requires an in-depth understanding of their specific context and experience with their management of MetS. In order to understand specific contexts and experiences, we explored gender-based differences in factors associated with cumulative probability of MetS resolution among newly diagnosed patients.

## Materials and methods

### Study design

This was a secondary analysis of a previous clinical trial study which explored the effectiveness of dietary counseling [22]. The primary study, which provided the data, is registered in the Clinical Research Information Service by Korea Centers for Diseases Control and Prevention, Republic of Korea (registration number: KCT0000275 and KCT 0000446). The primary study was approved for middle-aged (n = 500) and older (n = 360) adults. To explore gender-differences in MetS resolution in all adults, we used data of the extended study population of the primary research (both intervention and control groups) in our analysis. Briefly, otherwise healthy men and women aged 40–80 years who were diagnosed with MetS were recruited at 16 health facilities in 2010. MetS was defined by the NCEP-ATP III criteria for MetS and the Asia-Pacific standard for abdominal obesity [23]. According to the criteria, those who have ≥ 3 of the five components of MetS are defined to have MetS. We excluded patients who agreed to participate but provided incomplete information for core study questions in the survey and who had not provided signed consent for the study. We further excluded patients who were in treatment or on medication for MetS-related health conditions such as hypertension, dyslipidemia, and diabetes because any medical treatment for MetS-related conditions can be a confounder as it is associated with both exposure and outcome [24]. Finally, 637 participants were included for the health examination and survey and followed up for multiple post assessments for around 12 months (S1 Fig). The primary outcome of this study was MetS resolution, which was defined as having <3 MetS components within 12‐month of the treatment period. This study is a secondary analysis of anonymized data from the extended population of a primary study, which obtained informed consent from participants. This study was exempted from review by the Institutional Review Board (IRB) of Seoul National University.

### Measurement of MetS components

For all participants, 5 MetS components were assessed at baseline and every visit which was within a 2–3 months-interval. Trained research assistants performed clinical and anthropometric measurements according to a standardized protocol. Blood pressure was measured with an automatic digital sphygmomanometer (TM-2655P, A&D, Tokyo, Japan). Measurements were made after participants being seated for at least 5 min in a chair with both feet on the floor. Determination of blood pressure is based upon the mean of two measurements separated by at least 2 min. Waist circumference was measured with a tape spring-tension measure at a level midway between the lowest rib and the iliac crest. Fasting blood samples were drawn and analyzed at KAHP laboratories. Fasting glucose, triglycerides and HDL-cholesterol were measured using HITACHI 7600–110 (HITACHI, Tokyo, Japan). Sociodemographic characteristics of the participants including age, education level, marital status, employment status, monthly income, social support, self-rated health, and average hours of sleep were recorded at baseline by trained interviewers.

### Covariates

To consider potential confounding effect in the association between gender and MetS resolution, we included the following covariates in the model: age group (40s, 50s, 60s, and 70s), level of education (middle or lower, high school, college or university), household income (<$2,200, $2,200–5,500, and >$5,500), body mass index (BMI; <25, 25–30, and >30), current employment, smoking, alcohol drinking, living with partner, enrollment in health education classes, social support, sleep hours, moderate exercise (more than once a week), number of initial MetS components, and initial HbA1c (%).

### Statistical analysis

Baseline sociodemographic and MetS characteristics were compared between men and women using the chi-square test for discrete variables and the *t*-test for continuous variables. Cox proportional hazard model including all variables was applied to calculate adjusted hazard ratio (HR) of MetS resolution. To identify gender-based difference in the association between variables and cumulative probability of MetS resolution, we tested interactions by gender for all covariates. All statistical analyses and plotting were performed using the R statistical software package (R Version 3.2.1).

## Results

Among the 637 patients, 64.5% were women and the majority were aged between 50 and 60 years (Table 1). Within the observational period, 47.6% (95% confidence interval (CI): 43.6, 51.5%) of participants achieved MetS resolution. Resolution rate was not different by gender (44.7% of men and 49.1% of women). Compared by gender, women participants were more likely to be older, have a lower household income, and doing moderate intensity exercise regularly. Men are more likely to be employed, more educated, currently smoking and drinking, compared to women. Although women were statistically significantly older, the observed difference was 2.8 years which was not considered clinically meaningful.

**Table 1:** Comparison of baseline characteristics stratified by gender (n = 637).

	Men (n = 226)	Women (n = 411)	P for difference
**Age, mean (years)**	55.6 ± 9.1	58.4 ± 7.8	< 0.001
**Education, n (%)**			
**Middle school or lower**	50 (22.2)	216 (53.7)	< 0.001
**High school**	86 (38.2)	124 (30.8)	
**College or University**	89 (39.6)	62 (15.4)	
**Household income, n (%)**^**a**^			
**< $2,200/month**	65 (29.7)	208 (53.6)	< 0.001
**$2,200–5,500/month**	111 (50.7)	132 (34.0)	
**> $5,500/month**	43 (19.6)	48 (12.4)	
**Body mass index (kg/m**^**2**^**)**	27.2 ± 2.4	26.4 ± 2.8	0.002
**Paid working, n (%)**	142 (63.7)	126 (31.3)	< 0.001
**Current smoking, n (%)**	68 (30.1)	12 (2.9)	< 0.001
**Current drinking, n (%)**	170 (75.2)	148 (36.0)	< 0.001
**Living with partner, n (%)**	203 (89.8)	307 (75.1)	< 0.001
**Enrollment in health education classes, n (%)**	119 (52.7)	229 (55.7)	0.509
**Social support, n (%)**	15 (6.8)	46 (11.4)	0.085
**Average sleep hours**	6.4 ± 1.4	6.4 ± 1.4	0.914
**Moderate exercise (≥ once a week), n (%)**	48 (53.3)	41 (25.5)	< 0.001
**Number of initial MetS components**	3.26 ± 0.39	3.28 ± 0.51	0.646
**Initial HbA1c (%)**	8.80 ± 5.14	8.91 ± 4.44	0.194

^a^Household income was converted from Korean Won based on the mean currency exchange rate in 2010. Homogeneity between men and women was tested using Wilcoxon rank sum test (continuous variables) and Chi-squared test (categorical variables).

**Fig 1:**
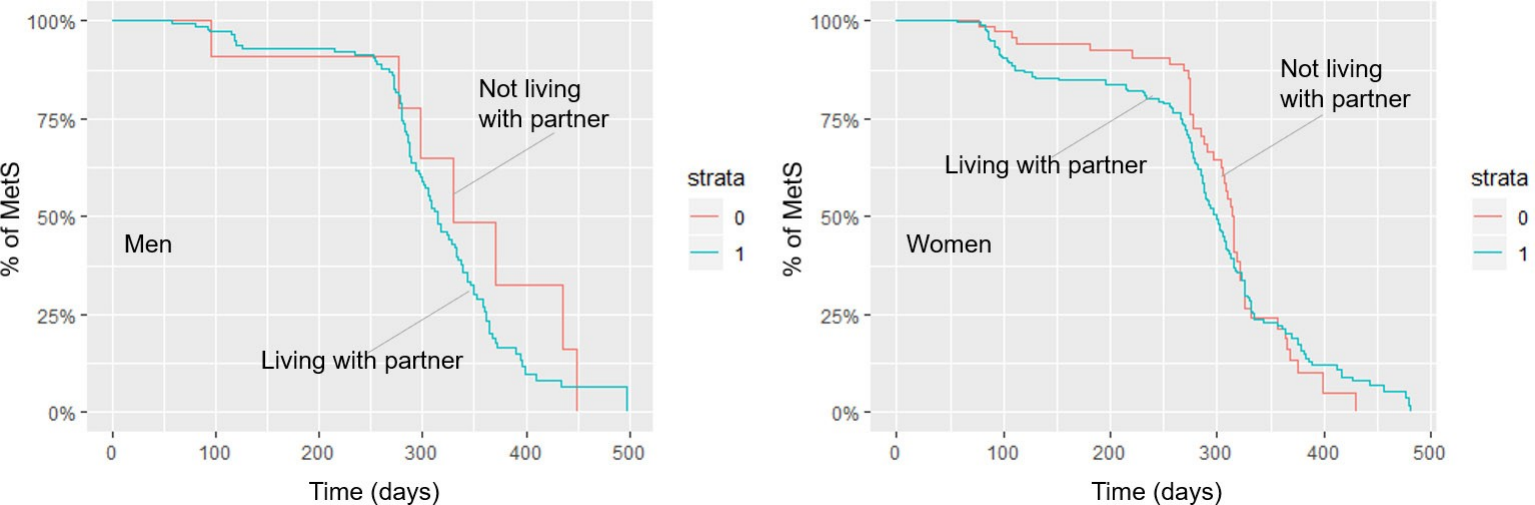
Time to metabolic syndrome resolution (days) stratified by living with partner (n = 637), (A) Men and (B) Women.

Patterns of change in MetS components during the study period differed between men and women (S2 Fig). Women showed a more profound decrease of hypertension and low HDL-cholesterol than men. In the multivariable analysis for the cumulative probability of MetS resolution within the study period, we observed multiplicative interactions by gender for current employment and sleep hours (S1 Table). Subgroup analysis by gender revealed different patterns of association between men and women (Table 2). Low household income (Hazard ratio = 2.62, 95% confidence interval: 1.13–6.08) and current employment (2.29, 1.26–4.13) were associated with higher cumulative probability of MetS resolution in men than in women. For women, however, longer sleep hours (1.18, 1.04–1.34) and living with a partner (1.58, 1.06–2.35) were positive predictors of MetS resolution within 12 months of observation (Fig 1). Overweight (0.63, 0.44–0.89), but not obesity, was associated with lower cumulative probability of resolution. Level of education, current smoking, alcohol use, enrollment in health education classes, social support, and initial HbA1c were not associated with time to resolution during the observation period for both men and women.

**Table 2:** Hazard ratios for time to achieving metabolic syndrome resolution during the observation period (n = 637).

	Men (n = 226)			Women (n = 411)		
	HR^a^	Lower	Upper	HR^a^	Lower	Upper
**Age groups**						
**40s**	1.00 (Reference)			1.00 (Reference)		
**50s**	1.20	0.61	2.33	1.06	0.64	1.75
**60s**	1.95	0.87	4.40	**2.33**	**1.25**	**4.36**
**70s**	**5.63**	**1.46**	**21.74**	1.94	0.84	4.48
**Education**						
**≤ Middle school**	1.43	0.63	3.23	0.63	0.37	1.05
**High school**	0.84	0.47	1.50	0.71	0.43	1.19
**≥ College or higher**	1.00 (Reference)			1.00 (Reference)		
**Household income**						
**< $2,200/month**	**2.62**	**1.13**	**6.08**	1.33	0.74	2.40
**$2,200–5,500/month**	1.27	0.65	2.44	0.92	0.52	1.62
**> $5,500/month**	1.00 (Reference)			1.00 (Reference)		
**Body mass index**						
**<25 kg/m**^**2**^	1.00 (Reference)			1.00 (Reference)		
**25–30 kg/m**^**2**^	0.86	0.47	1.60	**0.63**	**0.44**	**0.89**
**> 30 kg/m**^**2**^	1.14	0.48	2.70	0.97	0.53	1.79
**Current employment**	**2.29**	**1.26**	**4.13**	0.82	0.57	1.19
**Current smoking**	0.53	0.27	1.04	0.63	0.25	1.61
**Current alcohol drinking**	1.55	0.81	2.96	0.89	0.60	1.31
**Living with partner**	1.87	0.70	5.00	**1.58**	**1.06**	**2.35**
**Enrollment in health education classes, n (%)**	1.04	0.63	1.70	0.90	0.64	1.26
**Social support**	0.59	0.20	1.76	0.66	0.40	1.09
**Sleep hours**	0.87	0.75	1.02	**1.18**	**1.04**	**1.34**
**Moderate exercise (≥ once a week),**	**0.53**	**0.32**	**0.90**	0.83	0.56	1.23
**Number of initial MetS components**	**0.65**	**0.53**	**0.79**	**0.66**	**0.58**	**0.75**
**Initial HbA1c (%)**	0.99	0.92	1.07	0.99	0.94	1.04

HR, hazard ratios; MetS, metabolic syndrome.

^a^Estimates with P values < 0.05 are shown in bold.

## Discussion

We observed an interaction by gender in the association between clinical characteristics and MetS resolution within 1 year. There were gender-based differences in predictors for cumulative probability of resolution among newly diagnosed MetS patients. Household income and current employment were positive predictors in men while longer sleep hours and living with a partner were positive predictors in women. Although men and women had substantially different general characteristics, the MetS resolution rate among them was similar. This finding would provide empirical evidence for gender-based differences in prognostic predictors of MetS resolution within the first year of diagnosis.

Gender differences in the prediction of the prognosis of MetS have been noted in previous studies. Although the findings were inconsistent due to context and study settings, responses to intervention or treatment of MetS have been different between men and women in some of the studies. Metformin did not have effect over placebo in women but not in men in a study of intensive lifestyle intervention and metformin therapy [25]. In a prospective study of middle-age adults, C-reactive protein (CRP) was a significant predictor for the development of MetS in women [26]. On the other hand, in the study of MetS patients undergoing bariatric surgery, women were more likely to resolve the condition in 12 months compared to men after controlling for postoperative weight loss [27]. Yet the biological or sociocultural mechanism of this gender-difference in predictive factors for MetS prognosis remains to be elucidated, it would suggest gender-specific approach would be necessary in treatment of MetS patients.

The modification effect by gender of the patient in the association between living with partner and time to MetS normalization is noteworthy. In general, marriage has been reported to be advantageous to a better metabolic profile [28, 29]. Several studies have demonstrated gender-based difference in the association between marriage and better health. A Taiwanese study showed that having a stable marriage decreased the risk of cardiovascular disorders for middle-aged women, but not for men and young women [30]. The gender-based difference is postulated to be mediated by differential marital distress in these gender and age groups. In a study of English middle-aged couples, association between marital distress and the MetS remained significant for women but not for men [31]. In a study of middle-aged women, the risk of MetS in widowed and martially dissatisfied women was similar to that in divorced or widowed women, but higher than that in martially satisfied [29]. In the context of middle-aged Korean women, with historical gender inequality in social activities and employment, living with marital partner would have provided a more stable socioeconomic status compared to single women. On the other hand, this might have been not significant benefit for married middle-aged men. This finding requires validation by future studies of countries with a similar socioeconomic status.

This study had several limitations: First, as a small group of retrospective cohorts in primary health clinics, our study population may not be representative for general population especially for men. And the prevalence of severe obesity (defined as BMI ≥35 kg/m^2^) was 1% in our population which is far lower than that of other studies. Given the higher incidence of MetS in women in previous reports of Asian population [8–11] and very low prevalence of severe obesity in Korean adult ≥19 years old (0.2–0.5%) [32], we believe the findings of our study population reflect general characteristics of Korean adults. In addition, general characteristics of study participants such as mean age at the time of diagnosis and number of MetS components, are largely similar to those of previous studies, the finding of our study would still have clinical implications applicable to other populations. Second, objective measurement of individual adherence to health provider’s advice for diet or exercise was not available. Intermediate outcome measures for individual compliance to lifestyle modification would provide more explanation to the causal relationship between the baseline social factors and MetS resolution. We believe this study minimized the residual confounding effect caused by different adherence or compliance by including enrollment in health education classes and self-reported moderate exercise as covariates.

## Conclusions

There was gender-difference in predictors of MetS resolution within 12 months of follow-up. These findings will facilitate further exploration on gender-specific approach for more effective intervention of MetS.

## Supporting information

S1 Fig Final selection of the study population. (DOCX)

S2 Fig Evolution of MetS components in the study population. (DOCX)

S1 Table Hazard ratios for achieving resolution of metabolic syndrome during the observation period (N = 637). (DOCX)

S1 Data (CSV)
