# Supplementary figures and images for: Gender-differences in predictors for time to metabolic syndrome resolution: A secondary analysis of a randomized controlled trial study
Source: PLoS One. 2020 Jun 25;15(6):e0234035. doi: 10.1371/journal.pone.0234035 (PMC7316247; doi:10.1371/journal.pone.0234035)

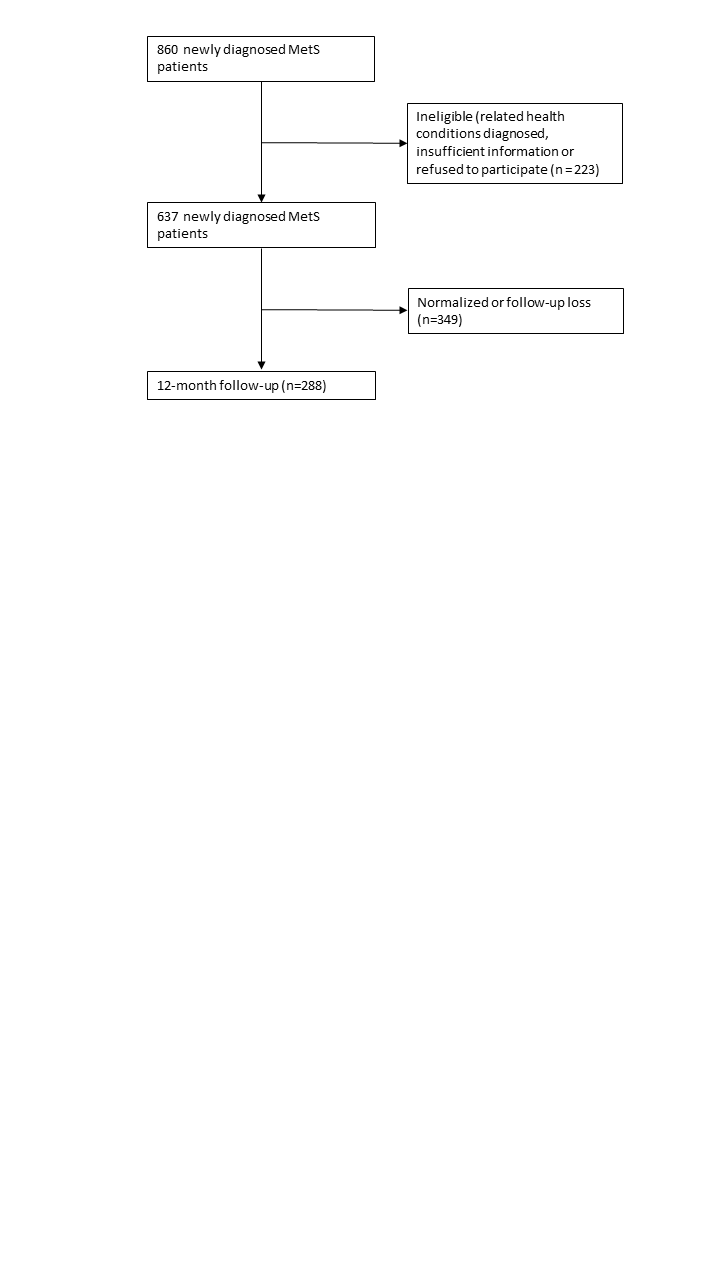


Supplementary Fig 1. Final selection of the study population

Supplement: S1 Fig — (DOCX) [file pone.0234035.s001.docx]
