# Supplementary material for: Gender-differences in predictors for time to metabolic syndrome resolution: A secondary analysis of a randomized controlled trial study
Source: PLoS One. 2020 Jun 25;15(6):e0234035. doi: 10.1371/journal.pone.0234035 (PMC7316247; doi:10.1371/journal.pone.0234035)
Supplement: S2 Fig — (DOCX) [file pone.0234035.s002.docx]

1. Men (n=226)
2. Women (n=411)

Supplementary Fig 2. Evolution of MetS components in the study population.
