# Supplementary material for: Gender-differences in predictors for time to metabolic syndrome resolution: A secondary analysis of a randomized controlled trial study
Source: PLoS One. 2020 Jun 25;15(6):e0234035. doi: 10.1371/journal.pone.0234035 (PMC7316247; doi:10.1371/journal.pone.0234035)
Supplement: S1 Table — (DOCX) [file pone.0234035.s003.docx]

Supplementary Table 1. Hazard ratios for achieving resolution of metabolic syndrome during the observation period (N = 637)

|  | Coefficient | se | P |  |  | Coefficient | se | P |
| --- | --- | --- | --- | --- | --- | --- | --- | --- |
| Age groups |  |  |  |  | Age groups |  |  |  |
| 40s |  |  |  |  | 40s |  |  |  |
| 50s | 0.11 | 0.20 | 0.595 |  | 50s | 0.05 | 0.20 | 0.819 |
| 60s | 0.81 | 0.24 | 0.001 |  | 60s | 0.75 | 0.23 | 0.001 |
| 70s | 0.88 | 0.34 | 0.010 |  | 70s | 0.96 | 0.34 | 0.004 |
| Education |  |  |  |  | Education |  |  |  |
| ≤ Middle school | -0.36 | 0.21 | 0.086 |  | ≤ Middle school | -0.29 | 0.21 | 0.162 |
| High school | -0.22 | 0.18 | 0.239 |  | High school | -0.18 | 0.18 | 0.312 |
| ≥ College or higher |  |  |  |  | ≥ College or higher |  |  |  |
| Household income |  |  |  |  | Household income |  |  |  |
| < $2000/months | 0.46 | 0.23 | 0.047 |  | < $2000/months | 0.35 | 0.22 | 0.120 |
| $2000-5000/months | -0.03 | 0.21 | 0.877 |  | $2000-5000/months | -0.09 | 0.21 | 0.658 |
| > $5000/months |  |  |  |  | > $5000/months |  |  |  |
| Men | -0.57 | 0.23 | 0.013 |  | Men | 1.63 | 0.60 | 0.007 |
| Body mass index |  |  |  |  | Body mass index |  |  |  |
| <25 kg/m^2^ |  |  |  |  | <25 kg/m^2^ |  |  |  |
| 25-30 kg/m^2^ | -0.36 | 0.14 | 0.012 |  | 25-30 kg/m^2^ | -0.39 | 0.14 | 0.007 |
| > 30 kg/m^2^ | -0.10 | 0.24 | 0.684 |  | > 30 kg/m^2^ | -0.07 | 0.24 | 0.759 |
| Current employment | -0.18 | 0.18 | 0.300 |  | Current employment | 0.08 | 0.15 | 0.596 |
| Current smoking | -0.56 | 0.27 | 0.034 |  | Current smoking | -0.26 | 0.26 | 0.316 |
| Current alcohol drinking | 0.02 | 0.16 | 0.919 |  | Current alcohol drinking | -0.05 | 0.15 | 0.762 |
| Living with partner | 0.50 | 0.18 | 0.005 |  | Living with partner | 0.53 | 0.18 | 0.003 |
| Participation to diet counseling | -0.07 | 0.14 | 0.628 |  | Participation to diet counseling | -0.05 | 0.14 | 0.738 |
| Social support | -0.20 | 0.22 | 0.357 |  | Social support | -0.35 | 0.23 | 0.124 |
| Sleeping hours | 0.02 | 0.04 | 0.653 |  | Sleeping hours | 0.17 | 0.06 | 0.007 |
| Moderate exercise ≥ 1/week | -0.31 | 0.15 | 0.042 |  | Moderate exercise ≥ 1/week | -0.26 | 0.15 | 0.076 |
| Number of initial MetS components | -0.38 | 0.05 | 0.000 |  | Number of initial MetS components | -0.40 | 0.05 | 0.000 |
| Initial HbA1c (%) | 0.00 | 0.02 | 0.825 |  | Initial HbA1c (%) | 0.00 | 0.02 | 0.937 |
| Men✕Current employment | 0.72 | 0.29 | 0.015 |  | Men✕sleep hours | -0.30 | 0.09 | 0.002 |
